# A qualitative assessment of an abstinence-oriented therapeutic community for prisoners with substance use disorders in Kyrgyzstan

**DOI:** 10.1186/s12954-017-0168-8

**Published:** 2017-07-10

**Authors:** Lyuba Azbel, Julia Rozanova, Ingo Michels, Frederick L. Altice, Heino Stöver

**Affiliations:** 1 London School of Hygiene & Tropical Medicine, Keppel St, Bloomsbury, London, WC1E 7HT UK; 2 Section of Infectious Diseases, Department of Medicine, Yale University School of Medicine, 135 College Street, Suite 323, New Haven, CT 06511 USA; 3 Institute for Addiction Research, Frankfurt University of Applied Sciences, Fachbereich 4: Soziale Arbeit und Gesundheit Nibelungenplatz 1, 60318 Frankfurt, Germany; 4 Centre of Excellence of Research in AIDS (CERiA), University of Malaya, Kuala Lumpur, Malaysia; 5 Division of Epidemiology of Microbial Diseases, Yale University School of Public Health, 135 College Street, Suite 323, New Haven, CT 06511 USA; 6 Central Asia Drug Action Programme (CADAP), Federal Ministry of Health, Friedrichstr. 108, Berlin, Germany

**Keywords:** Prisons, Kyrgyzstan, Substance use disorders, Therapeutic community, Opioid agonist treatment, People who inject drugs

## Abstract

**Background:**

Kyrgyzstan, where HIV is concentrated in prisons and driven by injection drug use, provides a prison-based methadone maintenance therapy program as well as abstinence-oriented therapeutic community based on the 12-step model called the “Clean Zone.” We aimed to qualitatively assess how prisoners navigate between these treatment options to understand the persistence of the Clean Zone despite a lack of evidence to support its effectiveness in treating opioid use disorders.

**Methods:**

We conducted an analysis of policy documents and over 60 h of participant observation in February 2016, which included focus groups with a convenience sample of 20 therapeutic community staff members, 110 prisoners across three male and one female prisons, and qualitative interviews with two former Clean Zone participants. Field notes containing verbatim quotes from participants were analyzed through iterative reading and discussion to understand how participants generally perceive the program, barriers to entry and retention, and implications for future treatment within prisons.

**Results:**

Our analyses discerned three themes: pride in the mission of the Clean Zone, idealism regarding addiction treatment outcomes against all odds, and the demonization of methadone.

**Conclusion:**

Despite low enrollment and lack of an evidence base, the therapeutic community is buttressed by the strong support of the prison administration and its clients as an “ordered” alternative to what is seen as chaotic life outside of the Clean Zone. The lack of services for Clean Zone patients after release likely contributes to high rates of relapse to drug use. The Clean Zone would benefit from integration of stabilized methadone patients combined with a post-release program.

## Background

Throughout the world, and specifically within Eastern Europe and Central Asia (EECA), incarceration, substance use disorders (SUDs), and HIV are inextricably linked [1]. The region’s proscriptive and punitive drug policies [2] concentrate individuals with past or current history of injection drug use within the criminal justice system. Kyrgyzstan is experiencing a rapidly expanding HIV epidemic concentrated among people who inject drugs (PWID), primarily with heroin. Both HIV prevalence and within-prison drug injection remain high [3]. Without utilization of evidence-based HIV prevention interventions, these individuals often engage in high risk-taking behaviors that are likely to result in transmission of blood-borne infections to others in prison and community settings. Furthermore, once prisoners are released into the community, those who continue to use drugs are more likely to experience adverse health consequences and be re-incarcerated, establishing a cycle of imprisonment and release. In the absence of policies that favor community treatment over incarceration, the criminal justice system should be harnessed as a means to curbing the intersecting epidemics of mass incarceration of PWID, HIV, and substance use disorders.

There are few evidence-based treatments available in prisons worldwide, where care often falls below community standards [4, 5]. Unlike in most countries, Kyrgyzstan is one of eight countries that provide all 15 internationally recommended HIV prevention strategies in prison [6]. Specifically, it provides a range of health services for prisoners with SUDs including prison-based needle & syringe programs (PNSPs), opioid agonist therapies (OAT) using maintenance with methadone, and therapeutic communities including an abstinence-only prison facility. PNSPs markedly reduce needle sharing and HIV transmission [7–9]. OAT is internationally recognized as the most effective treatment for chronic opioid dependence as well as one of the most effective primary and secondary HIV prevention strategies available [10]. When continued after release, it also  significantly reduces post-release mortality [11]. Therapeutic communities are structured environments where behavioral treatment of substance use disorders promotes peer support to improve drug use and social outcomes, often involving drug-free facilities and 12-step Narcotics Anonymous programs [12]. Therapeutic communities are most effective in patients with polysubstance use disorders but fare poorly relative to OAT in patients with opioid use disorders [13]. In prison contexts, therapeutic communities show mixed results, with only short-term reductions in substance use and re-incarceration, especially without a sustained aftercare program post-release [14]. Indeed, in a recent study of newly released prisoners with opioid use disorders in Malaysia, those who had received 24 months of therapeutic community support during detention were significantly less likely (10 vs 40%; *p* < 0.001) to have remained free from opioids within 12 months as compared to those who received voluntary methadone maintenance treatment [15].

Despite the absence of data supporting the use of rehabilitation facilities based on the 12-step model, doctors, employers, and judges regularly refer patients with SUDs to these treatment programs. Why the model has popularity among providers and clients and how they perceive it to work even in the presence of suboptimal results remain to be explored. Kyrgyzstan provides a valuable case study as the only post-Soviet country to have such an abstinence-only treatment facility integrated into the criminal justice system. This independent unit embedded within a prison and known as the “Clean Zone” has provided a 12-step treatment model over the past 10 years but has not previously been independently assessed. Moreover, an understanding of its cultural significance may help shed light on the persistence of such programs despite the lack of corresponding evidence attesting to its effective treatment of SUDs. Such an assessment can help ensure a culturally appropriate solution to the future of 12-step models in EECA prisons, especially considering that Kyrgyzstan serves as a regional leader on HIV/AIDS prevention and treatment policies [16]. Given recent plans to expand the abstinence-only treatment throughout the country, this study aims to qualitatively assess how and why the Clean Zone is being supported in order to guide the development of appropriate programs in a region with an urgent need to harness the criminal justice system to treat SUDs and prevent HIV.

## Methods

Focus groups with therapeutic community clients and individual interviews with staff members were conducted in February 2016. These were complemented by a review of secondary data sources and policy documents on the implementation of the 12-step program and the “Clean Zone” prison in Kyrgyzstan.

### Description of the Atlantis and Clean Zone programs

In Kyrgyzstan, where the prison population is 7683 [17], 30.4% of prisoners report prior drug injection [5], with 86% of these having done so within prison [5]. Before introducing the “Clean Zone,” a 12-step rehabilitation program based on the Atlantis Model [18] was introduced and is now available in eight Kyrgyz prisons. These Atlantis programs differ in each facility by virtue of whether participants are housed privately from other prisoners. Entry is voluntary, but participation requires extensive participation in group therapy sessions and workshops with trained social workers for up to 18 months. Participants must sign a pledge to reject all psychoactive substances including medications to treat psychiatric or substance use disorders prescribed by physicians. Graduates of the Atlantis program may opt to return to their prison units or transfer to the Clean Zone. Eligibility to transfer to the Clean Zone is stringent and requires a review of their participation in Atlantis and an interview with the Director of the Clean Zone to ensure their commitment to a “drug-free” lifestyle. The Clean Zone concept emerged from guidance from Pawel Moczydlowski, a Polish criminal justice expert, who saw it as a space for the prison administration to regain control of prisons overrun by a strong prison subculture powered by an illicit drug trade. The aims of the Clean Zone were to reduce recidivism, decrease the drug trade and drug use within prison, and prevent transmission of infectious diseases. Kyrgyzstan’s Clean Zone opened in 2010 and provides markedly improved living conditions and a number of vocational programs for clients.

### Study questions

The aim of the study was to conduct a qualitative and independent assessment of the functioning of the “Clean Zone” and provide recommendations to prison administrators and funders. In doing this, we combined principles of inductive and deductive reasoning [19]. Through qualitative interviews with various stakeholders, we sought to answer the following study questions that addressed both pre-determined codes and themes that emerged during fieldwork:

1. What are the barriers and facilitators to entry and retention in the Clean Zone program?
2. What information is available on the effects of this program on the treatment of SUDs among clients within prison and after release?
3. Is the program being conducted with an efficient use of resources?
4. How is the “Clean Zone” program viewed by both prisoners and staff and how does this compare to attitudes regarding OAT in treating opioid use disorders?
5. How can the program be improved to better meet the health needs of prisoners with SUDs?

### Study sites and sampling of study participants

Three researchers (FLA, LA, JR) visited four sites, including the Clean Zone and three Atlantis programs over one week in February 2016 and spent over 60 h conducting participant observation as well as focus groups with a convenience sample of participants (110 currently incarcerated prisoners across four prisons—three male and one female—two released former Clean Zone participants who currently work in local NGOs that assist prisoners upon release) and staff members (*N* = 20) of the therapeutic community. The choice of the Atlantis sites, made by the prison administration, was motivated by their proximity to the capital city of Bishkek. Of the three Atlantis centers visited, one was cordoned off from the rest of the prison, creating an isolated environment similar to the Clean Zone. The Clean Zone is an independent unit located on the territory of Prison 31. Eligible study participants included prisoners (participants of the Atlantis and Clean Zone programs), staff (Atlantis and Clean zone), prison administrators, and released Clean Zone participants (NGOs).

### Data collection

Both clients and staff were involved in individual in-depth interviews and focus groups. The Clean Zone staff also provided administrative records that were reviewed. Additional observation of the Atlantis and Clean Zone environments were also conducted to better understand the treatment delivery context. This included sitting in during group 12-step meetings. When available and appropriate, we also employed analysis of policy documents (such as the care-provision protocols used by Atlantis staff members). Focus groups and interviews were advertised to a group of potential participants. It was made clear that participation would not affect their stay in the prison, all questions would be voluntary, and all responses would be anonymous.

A topic guide was used for each focus group and individual interview, which was developed iteratively through a combination of lines of questioning from emerging findings and pre-conceived markers of program evaluation literature [20]. The guides covered day-to-day experiences and history within the programs, attitudes toward addiction treatment, barriers to entry and retention, social support, and anticipated challenges after release from prison. With Atlantis clients, we led two focus groups at two men’s programs and one focus group at the women’s program, and with the Clean Zone clients, we conducted one focus group. Individual in-depth interviews were conducted with Atlantis and Clean Zone staff which included the director of the program and social workers from all three prisons and two “graduates” of the Clean Zone program who were already released from prison and working with NGOs that provide services to prisoners.

Upon consultation with the prisons department, focus groups were not audio-recorded to ensure participants’ comfort and openness of the discussion of sensitive topics as well as the expression of diverse opinions within the group. This was also relevant as one of the authors did not speak Russian and thus interactions between the researchers and the participants were mediated by interpretation provided by the other two researchers. All focus groups lasted about 90 min and interviews lasted 30–45 min. Instead of audio-recording, extensive field notes were taken by three of the authors during the focus groups and participant observation. Field notes included verbatim quotes of particular salience from participants. These interviews were supplemented by informal conversations we had with our colleagues in Kyrgyzstan and in Ukraine—such as the staff of the non-governmental organization AIDS Foundation East-West, the NGO “Ranat” in Bishkek, and the Ukrainian Institute for Public Health Policy. These meetings gave us a sense of the public profile that the Clean Zone and Atlantis have.

### Analysis

First, we assessed the effectiveness of the program based on analysis of policy documents and interviews with study participants (study questions 1–3). Based on scant evidence attesting to the long-term effectiveness of therapeutic communities in treating SUDs [21, 22] and deficiencies in the performance of the Clean Zone, we then sought to understand the motivation for continued government support of abstinence-based treatment even where methadone maintenance therapy was readily available (study question 4).

Extensive field notes were analyzed to draw conclusions from unstructured observations [23]. Immediately after the focus groups, the three authors who conducted the fieldwork debriefed to share observations and field notes that were taken during debriefing sessions. Field notes included everyday lived experiences of both prisoners and staff members, their understanding of addiction and addiction treatment, their healthcare needs, and their attitudes toward methadone- and abstinence-based addiction treatment programs as well as observations of the participants’ interactions with each other and staff, comfort level, and living conditions. As we conducted our unstructured observations as a small group of researchers with diverse backgrounds (a public health epidemiologist currently trained as a medical sociologist, a sociologist trained as a qualitative health researcher, and a senior clinician scholar trained in infectious diseases and addiction treatment), we capitalized on our interdisciplinary diversity in our analysis of fieldwork data. Our analysis included iterative reading and discussing our field notes and combing them for recurring themes that would most accurately describe and represent the lived experiences of participants as they related to addiction treatment in Kyrgyz prisons.

Throughout our fieldwork and analyses, we discussed how our professional and academic backgrounds shaped our reflective “gaze” when encountering participants [24]. Through this process, we acknowledged the epistemological limits of our comprehension of the participants’ lived experiences and aimed to push these limits by triangulating our collective understanding embedded in our diverse experiences and training [25]. Most importantly, by challenging our emerging findings from different disciplinary perspectives and combing our data for possible negative cases, we strove to maintain a balance between closeness, distance, and honesty in our representation of participants’ lived experiences and concerns [26].

## Results

Prisoner participants were predominantly male (81%, mean age 37) whereas staff participants were predominantly female (67%).

With regard to the study questions, we found the following:

*How effective is the Clean Zone and the Atlantis program at treating SUDs?*

1. What are barriers and facilitators to entry and retention in the program?

Nearly all individuals within the Atlantis program did not advance to the Clean Zone for various reasons: (1) insufficient time left on their sentence; (2) individual preferences not to participate, primarily due to an understanding of the Clean Zone being a space with restricted movement and stringent rules; (3) not deemed eligible by Clean Zone staff who assess whether they would be optimal candidates or not; (4) many do not complete the Atlantis program and therefore are not eligible; and (5) stigma of being associated as “loyal” to the prison administration rather than to other prisoners.

2. What information is available on the effects of this program on the treatment of SUDs among clients within prison and after release?

This conclusion is verified and supported by the presence of prisoners in the colonies (especially in the Atlantis program) who have completed the Clean Zone previously and described their relapse trajectory in which they are released from prison to the community, but in the absence of employment, housing, and social support, nearly all participants relapse and this relapse occurs almost immediately after release. Furthermore, the data collected by the Clean Zone staff also suggest many patients (close to 30% in total over the 5 years of program operation) drop out of the program either by refusing therapy or breaking the rules of the program.

3. Is the program being conducted with an efficient use of resources?

There are currently a maximum of 12 to 24 available slots in each Atlantis program at eight prisons, yet in almost all instances, they were not full. Thus, the 100-person capacity of the Clean Zone program, with 19-funded clinical and custodial staff, remains considerably underutilized, with the maximum number of participants in the program at any one time being 45. Staff reported marked difficulties in being able to recruit a sufficient number of participants from the Atlantis program into the Clean Zone. 

*Why does support for the Clean Zone persist despite the lack of evidence base?*

4. How is the “Clean Zone” program viewed by both prisoners and staff and how does this compare to attitudes regarding OAT?

Three themes emerged that provide insight into the cultural significance of the Clean Zone program.

### Theme one: pride in the mission

The staff of the Clean Zone and Atlantis, as well as the Clean Zone clients, presented with an omnipresent sense of pride toward the goals of the work they were set to accomplish. The staff seemed to be sincere enthusiasts who deeply enjoy their work with opioid-dependent prisoners; they were knowledgeable about and experienced with the 12-step program and had also undergone various training programs and seminars (as testified by the numerous diplomas and certificates displayed on the walks of their offices) to extend their appreciation and understanding of how the 12-step program works.

The director of the Clean Zone seemed to exude a matron-like concern for the well-being of the clients and the performance of her duties. When she referred to her clients, she used the word *rebiata*, i.e., boys, which, in the Russian language, has affectionate and slightly patronizing undertones used when a senior person makes a reference to a junior person under their wing. Furthermore, the director of the Clean Zone performed the functions of the matron by advocating for her charges toward various organizations like NGOs, trying to raise funds to cover the expenses of keeping the facilities tidy, well-stocked with food supplies, warm, and in a good state of repair in winter. A former colonel of the Kyrgyz police told us a story of how the director of the Clean Zone went to great lengths to have a heating unit replaced in the facilities in the late fall a year ago to ensure the clients have comfortable living conditions. It is our impression that, to the clients, many of whom had no close family, the director of the Clean Zone serves as a parental figure, providing ongoing care and a sense of support. Also, she performed the parental role of providing a strict moral code of conduct, and her ardent belief in the values she was bringing forward (including not only sobriety as such but also the idea of a productive and socially beneficial lifestyle associated with sobriety) enhanced the legitimacy of these values in the minds of the clients.

Professional pride on the part of the staff was matched by the prisoners’ enthusiasm toward the goals of sobriety and social recovery. There was a particularly exuberant contingent among the clients who stated that they appreciated feeling a part of a community and having support of the other clients who were responsible for each other. They stated that they also had a responsibility to report on others who were using drugs or not behaving “adequately.” The Clean Zone director later confirmed this, saying that surprise drug testing is carried out in the facility. The social support found in this program was given a lot of value, since many of the participants lacked this sort of support in the community.

### Theme two: idealism against all odds

A strong idealism was reflected in the strict adherence to order and the goals of sobriety in the face of a lack of evidence for the continued effect of this program after release. Given the challenges of post-release life and a lack of support after release, changes for sustained sobriety were bleak. Yet, staff and prisoners alike set their aim at sobriety which was understood as complete abstinence from any substance use, including legally prescribed medications like methadone, for the rest of their lives. Idealism was also visible through the prisoners’ ardent insistence on stating their own exceptionality and on how they felt being different, stronger, and more mature—both in comparison to the rest of their peers and in comparison to themselves prior to entering the Clean Zone. Each of them wanted to feel as though they were the exception, not the rule; if the rule was that a substance use disorder was a chronic and relapsing condition, each prisoner in the Clean Zone wished to assert themselves as being an exception to this process where relapse would not happen to somebody like themselves. To support this vision of their new, improved selves, during the focus group discussions, they shared stories how other, sicker, and/or older prisoners had given them a kind of a blessing to pursue sobriety and a decent life and how these other prisoners expressed trust in their abilities and will to accomplish this goal. Yet this idealism meant that Clean Zone clients segregated themselves from the rest of their extended primary peer group of other prisoners with a SUD not only spatially (by living in a separate facility) but also culturally, and cultivated a sense of moral superiority.

Simultaneously, they appreciated that the level of social support that was accessible to them through Atlantis programs and/or Clean Zone was unattainable upon release: many prisoners had no families and no home to return to and many worried about jobs and income. Only three former clients of the Clean Zone had jobs in NGOs and six had volunteer positions, with the others’ employment situation being uncertain. Only 67 released Clean Zone clients maintained contact with the Clean Zone and were known to be abstaining from using substances. In the face of such unlikely prospects for success, clients however persisted in their wish to be the exception and not the rule. While the staff and the prisoners appreciated that the lack of services upon release may present a challenge, they felt that the prisoners’ willpower would be the principal tool to keep them sober. When we spoke to the two graduates of the Clean Zone program, however, they recognized the need for a transitional program after release, saying “being confronted with reality on the other side, you’re faced with social problems, you continue using” and “if there’s no continuation of the Clean Zone, all the effort put into them here is useless.” They estimated that about 80% of inmates have no passports and no ability to get a passport, so that after release, they often return to illegal activities since a passport is necessary to find legal employment.

### Theme three: the demonization of methadone

The staff and the prisoners demonstrated a strong suspicion toward methadone which they did not view as a treatment, but, rather, demonized and feared it. This exposed a process of “othering” and seeing “us” (i.e., clients of the Clean Zone) versus “them” (i.e., those who are not strong enough, including those on methadone). Also, we detected a certain moral superiority in the prisoners’ discourse: for example, one prisoner in the Clean Zone during the focus group recounted a story of how an older prisoner who had taken him under his wing had instilled in him a fear of methadone as something he had taken but that is best avoided. The prisoner felt that “all eyes were on him” as he was going to try and accomplish the goals of sobriety that his (older and more senior) fellow prisoners had not been able to achieve. Prisoners who had chosen the Clean Zone were viewed and felt themselves as more advanced on the way to recovery than methadone patients. The idea that methadone patients could potentially share Clean Zone or Atlantis resources (as the resources in the Clean Zone were underutilized since it was functioning well below its capacity) was rejected vehemently by prisoners and by the staff. Those on methadone were not considered sober or “clean” by participants or staff and methadone patients were not seen as being on the path to recovery. This perspective points to the understanding of methadone as not being a viable treatment and therefore irreconcilable with the goals of the Clean Zone.

## Discussion

This study is the first to evaluate and describe an abstinence-only program in a criminal justice setting of EECA. It is particularly important to understand the persistence of the “Clean Zone” program in Kyrgyzstan, where opioid-dependent prisoners choose between internationally recommended HIV prevention services—NSP and methadone maintenance treatment (MMT)—and an abstinence-only 12-step treatment. Our fieldwork explored patient and provider perspectives about the program as well as analyzed the performance of the Clean Zone. In light of a worldwide literature attesting to the limited efficacy of 12-step programs in treating opioid dependence as well as the poor resource allocation of the Clean Zone, the program is buttressed by both the prison administration and its participants. We argue that negative attitudes among OAT participants undermine the path to recovery for opioid addiction, relegating addiction treatment with OAT to an inferior position in comparison to abstinence-only programs, despite the international literature supporting it to be the most effective treatment for opioid addiction. Here, we explore the cultural value attributed to the Clean Zone program, particularly in relation to social standing within the prisoner subculture, which must be understood in order to proceed with the effective implementation of drug treatment in the Kyrgyz as well as other EECA criminal justice systems.

While no systematic research assessment of the Clean Zone’s effectiveness has been conducted to date, our interviews with staff and participants, as well as the data collected and shared with us by the Clean Zone staff, suggest that relapse to alcohol and drug use is high among the Clean Zone’s former patients. This is consistent with the overwhelming majority of evidence, which shows that 12-step programs have only a 5 to 15% success rate at abstinence from long-term opioid dependent persons, which is hardly better than no treatment at all [27, 28]. There is a glaring lack of services for Clean Zone patients after release from prison, which likely attributes to the patients’ high rates of relapse to drug use. The review of the literature suggests that any within-prison addiction treatment, especially those that use therapeutic community strategies like those in the Clean Zone and Atlantis, may only be effective if the continuity of services is available long-term and continue after release. Studies demonstrate that between 25 and 55% of clients of therapeutic communities relapse to drug use after 12 to 18 months [29]. The best evidence for addiction treatment in prisoners who do not receive OAT is with therapeutic communities where intensive treatment started inside the prison, is continued for 6 to 18 months after release and includes an array of vocational and employment services in the community [29–31]. Thus, the key concern is that the current Clean Zone has no aftercare activities. Women prisoners have no access to the Clean Zone, so they cannot continue the 12-step program beyond Atlantis. Even the Atlantis program in the female prison remains underutilized. Nonetheless, creating a special Clean Zone for women remains the prison administration’s priority—a questionable use of scarce resources. Women participants of the Atlantis program pointed to the lack of aftercare services as their main concern, including both assistance with finding stable transitional housing, employment, obtaining a passport, other legal documents, help with re-integration into the community, and social and psychological support. Dormitory facilities for released prisoners, which used to be available through local NGOs, are now almost inaccessible or closed due to the lack of funding.

Our analysis revealed that the Clean Zone program’s resources and capacity are highly underutilized. The Atlantis program—the only feeder pathway to the Clean Zone—acts as a bottleneck to the Clean Zone which is operating significantly under capacity. The perception among other prisoners that those who enter the Clean Zone are “traitors”—fleeing prisoner life to hide in the secluded Clean Zone for their offenses against the prisoner subculture—emerged as an especially strong driving force against entering the program. This lends weight to the argument that it is not the lack of effectiveness but, rather, the social standing among peers that plays a greater role in disincentivizing the Clean Zone. Further qualitative research should explore prisoner subculture in shaping decisions for the treatment of opioid dependence.

Despite the lack of evidence for recovery and the strong opposition to the program among the prisoner population in general, a strongly minded minority decides to take part in the Clean Zone. This idealism, as noted in research elsewhere within EECA [32], is common when housed in a protected environment. A study from Ukraine demonstrated that recovery from addiction and treatment with OAT were predominantly viewed as mutually exclusive processes [33]. Important from this study is that current prisoners exhibited higher optimism about changing their drug use, were less likely to endorse methadone, and reported higher intention to recover from their addiction compared to a similar group of recently released prisoners who were now in the community. A recent study by the the National Academies of Sciences, Engineering and Medicine [34] found that being prescribed methadone maintenance is not inconsistent with the ideals of recovery, which allows for new opportunities for treating opioid use disorders.

Our recommendations for the Clean Zone program are to:

### Recommendations

- Establish a transitional program for soon-to-be-released Clean Zone clients that continues 6 to 18 months after release, including shelter, social rehabilitation, linkages to employment, and preparation of legal documents. This can be accomplished at no additional costs by cost-shifting from the 100 bed prison unit to aftercare activities post-release.
- Create similar rehabilitation opportunities for methadone patients who are not using illegal substances. A Clean Zone for stable methadone patients would be equally beneficial to them, provide opportunities for transitional care, and address the negative and undermining attitudes of Clean Zone participants toward OAT. Such a strategy of integrating methadone patients into the underutilized Clean Zone should proceed in order to align goals for recovery for all patients who are not using illegal substances. 
- Remove the barriers to entering the Clean Zone in order to fill to capacity, which would minimally include stable methadone patients that have as their goal to remain off of illegal substances.

### Limitations

The data are limited by its cross-sectional assessment and non-representative nature. Using qualitative data can be useful for program development, however, especially as a quantitative assessment would not be reasonable here given the lack of worldwide literature to support abstinence-only programs. In this case, a qualitative study can better address the stakes that are involved in participants making choices for or against therapeutic community treatment or methadone maintenance for their opioid dependence.

## Conclusions

The low level of interest in the Clean Zone by prisoners themselves and high staff-to-client ratio suggest that, if the Clean Zone were to be continued, it would benefit from integration of stabilized methadone patients combined with a post-release aftercare program. The extraordinarily hostile and negative attitudes toward methadone maintenance treatment, however, persist among patients in the Atlantis program and the Clean Zone. This perception may, in part, have evolved from methadone being originally introduced as a harm reduction strategy alongside PNSPs, rather than as an evidence-based treatment for opioid use disorders. As such, methadone was viewed as a means to prevent HIV transmission, but not as an effective treatment for opioid addiction. The Clean Zone, based on within-prison therapeutic communities, harbors negative attitudes toward OAT, stating that individuals with addiction problems cannot be in recovery from their addiction as long as they take any form of psychoactive substance, including medications prescribed by a physician. Indeed, programs such as the Clean Zone run contrary to the 2013 understanding of substance use disorders that are chronic, recurring conditions. These conditions, while never cured, may be in early (3–12 months) or sustained (>12 months) remission or effectively treated using maintenance therapy with OAT. Moreover, SUDs may be qualified as “not treated” when someone is in a controlled setting like an inpatient addiction treatment unit, hospitalized, or within a criminal justice setting [35]. The hostility toward methadone observed among Atlantis and Clean Zone patients risks making the patients feel particularly desperate if they relapse, closing off any other treatment options since methadone is not considered as a viable option.

## Abbreviations

CADAP: Central Asia Drug Action Programme
EECA: Eastern Europe and Central Asia
MMT: Methadone maintenance treatment
NGO: Non-governmental organization
PNSPs: Prison-based needle & syringe programs
PWID: People who inject drugs
